# Nomogram and risk-score for predicting overall survival and risk stratification in patients with sarcomatoid non-small cell lung cancer: a multicenter study of 135 patients

**DOI:** 10.1186/s12890-025-03796-6

**Published:** 2025-07-09

**Authors:** Wenjian Tang, Yujin Yin, Chunju Wen, Shuhua Luo, Jinsheng Huang, Bo Lan, Yuan Kang, Zhiqiang Zhang, Zhongjian Liao, Zhen Wu, Qing Chen, Jiawang Wei, Jing Qiu, Xingting Qiu, Hua Chen, Ming Jia, Junyuan Zhong, Jianping Zhong

**Affiliations:** 1 Ganzhou Institute of Medical Imaging, Ganzhou Key Laboratory of Medical Imaging and Artificial Intelligence, Medical Imaging Center, Ganzhou People’s Hospital, The Affiliated Ganzhou Hospital of Nanchang University, 16th Meiguan Avenue, Ganzhou, 341000 P.R. China; 2 Department of Hematology, Ganzhou People’s Hospital, The Affiliated Ganzhou Hospital of Nanchang University, Ganzhou, 341000 China; 3 Department of Pathology, Ganzhou People’s Hospital, The Affiliated Ganzhou Hospital of Nanchang University, Ganzhou, 341000 China; 4 Department of Oncology, Ganzhou People’s Hospital, The Affiliated Ganzhou Hospital of Nanchang University, Ganzhou, 341000 China; 5 Department of Medical Imaging, First Affiliated Hospital of Gannan Medical University, Ganzhou, 341000 China; 6 Department of Nuclear Medicine, Ganzhou People’s Hospital, The Affiliated Ganzhou Hospital of Nanchang University, Ganzhou, 341000 China; 7 Department of Radiology, Southern Medical University Nanfang Hospital, Guangzhou, 510515 China

**Keywords:** Sarcomatoid carcinoma, Non-small cell lung cancer, Computed tomography, Prognosis, Nomogram

## Abstract

**Background:**

To explore the clinical data and CT findings associated with outcomes prognosis of patients with sarcomatoid non-small cell lung cancer (s-NSCLC).

**Materials and methods:**

In this retrospective study, s-NSCLC patients who underwent contrast-enhanced thoracic CT from January 2013 to June 2023 at three centers were enrolled. Clinicoradiological data, including sex, age, smoking status, tumor–node–metastasis (TNM) classification, tumor size, tumor location, calcification, vacuole/cavity, hydrothorax, low-attenuation area (LAA) ratio and peritumoral ground-glass opacity (GGO) or nodules were calculated. Clinical and CT findings associated with overall survival (OS) were evaluated by a multivariate Cox regression model.

**Results:**

A total of 135 patients with s-NSCLC in three centers were included. s-NSCLC patients were more likely to be elderly male smokers. The mean age and tumor size at diagnosis were 62 years and 5.8 cm, respectively. The median survival time of patients with s-NSCLC was 9 (95% CI: 7, 11) months. The 1-, 2- and 3-year OS rates of the s-NSCLC patients were 36.6%, 26.7% and 21.4%, respectively. s-NSCLC is often peripherally located (98/135, 70.4%). Calcification and vacuole/cavity were rare in s-NSCLC lesions. The hydrothorax was present in 36/135 (26.7%) s-NSCLC patients. The s-NSCLC lesions usually presented with LAA (87/135, 80.6%), and the median LAA ratio was 30.8% (interquartile range, IQR: 10.6%, 50.7%). In the multivariate Cox regression analysis, smoking status (hazard ratio, HR = 1.668 [95% CI: 1.040, 2.678]), tumor size (HR = 1.818 [95% CI: 1.167, 2.832]), peritumoral GGO or nodules (HR = 2.064 [95% CI: 1.090, 3.909]) and M stage (HR = 2.479 [95% CI: 1.476, 4.164]) were associated with increased mortality, whereas surgery (HR = 0.467 [95% CI: 0.274, 0.797]) was associated with decreased mortality. We constructed a nomogram for predicting the 1-, 2- and 3-year OS rates of s-NSCLC patients, and the AUCs were 0.867, 0.905 and 0.911, respectively. Risk-score = 0.512×smoking status + 0.598×tumor size + 0.725×peritumoral GGO or nodules+(-0.761)×surgery + 0.908×M stage.

**Conclusion:**

The multivariate Cox regression model, which includes factors such as smoking status, tumor size, peritumoral GGO or nodules, surgery and M stage, is helpful in predicting the OS and risk stratification of s-NSCLC patients.

## Introduction

Sarcomatoid non-small cell lung cancer (s-NSCLC), a rare histological variant accounting for approximately 0.4% of all pulmonary malignancies, is pathologically defined by the coexistence of epithelial and mesenchymal components [1, 2]. As a highly heterogeneous subtype, s-NSCLC is frequently diagnosed at an advanced stage, exhibits resistance to conventional chemotherapy and has a worse prognosis than other NSCLC subtypes, with a median overall survival (OS) of less than 12 months in most cases [1–7]. Given its rarity and clinical heterogeneity, identifying reliable pretreatment CT diagnosis and prognostic evaluation for s-NSCLC remain critical challenges in clinical oncology.

Several clinical factors have been investigated as potential predictors of survival in s-NSCLC patients. Clinical characteristics, including age, sex, tumor–node–metastasis (TNM) classification, surgery and chemoradiotherapy have been identified as potential risk factors associated with the OS of s-NSCLC patients [4, 7]. Thoracic CT is a noninvasive method for the routine detection of tumor lesions and is helpful for TNM classification during clinical management decisions. CT findings of s-NSCLC were first reported by Kim et al. [8], who reported that 8 of 10 patients had a low attenuation area (LAA). Research has consistently revealed that s-NSCLC often presents with LAA and peritumoral ground glass opacity (GGO) [9–11]. For postoperative s-NSCLC patients, LAA is the only CT finding that has been identified as an independent risk factor associated with OS [10, 11]. However, the CT findings of s-NSCLC associated with OS reported in previous studies were based on small sample sizes.

This study was based on multicenter data, aiming to develop a multivariate prognostic model for s-NSCLC. By systematically evaluating clinical characteristics and CT findings, we sought to create a robust predictive tool using Cox regression and nomogram methodologies. These approaches are helpful for risk stratification and prognosis evaluation for patients with s-NSCLC.

## Materials and methods

### Patients

This retrospective study was approved by the institutional review board (IRB no. TY-ZKY2022-045-01), and the requirement for written informed consent was waived.

The data of s-NSCLC patients were collected at 3 institutions (institution 1: Southern Medical University Nanfang Hospital; institution 2: Ganzhou People’s Hospital; institution 3: the First Affiliated Hospital of Gannan Medical University) from January 2013 to June 2023. The inclusion criteria were as follows: (1) the diagnosis was confirmed by light microscopic findings and immunohistochemistry (sarcomatoid components accounted for more than 10% of the tumor tissues and expressed both mesenchymal and epithelial immunohistochemical markers), and (2) CT examinations were performed before treatment. The exclusion criteria were as follows: (1) patients who underwent treatment before CT examination; (2) those with poor image quality or missing images and (3) those lost to follow-up.

Clinical data, including sex, age, smoking status and long-term follow-up, were reviewed and recorded by six of the authors (WT, YY, CW, BL, JH, ZZ). The tumors were classified and staged according to the 8th edition of the tumor-node-metastasis (TNM) classification.

### CT image acquisition

CT images were obtained with a 64-detector row CT scanner (Somaton Definition AS_+_; Siemens Healthineers); and a 256-row multidetector CT scanner (Revolution CT; GE Healthcare and Brilliance iCT; Philips Medical systems) at 3 institutions. The CT scan protocols were as follows: the tube voltage was 100–120 kV, the tube current was automatically adjusted, the matrix was 512 × 512, and the reconstructed slice thickness was 1.25 mm. Plain scan (PS), arterial phase (AP) and venous phase (VP) images were obtained. AP and VP scans were performed at 25 s and 60 s after contrast injection.

### CT scan interpretation

**Fig. 1:**
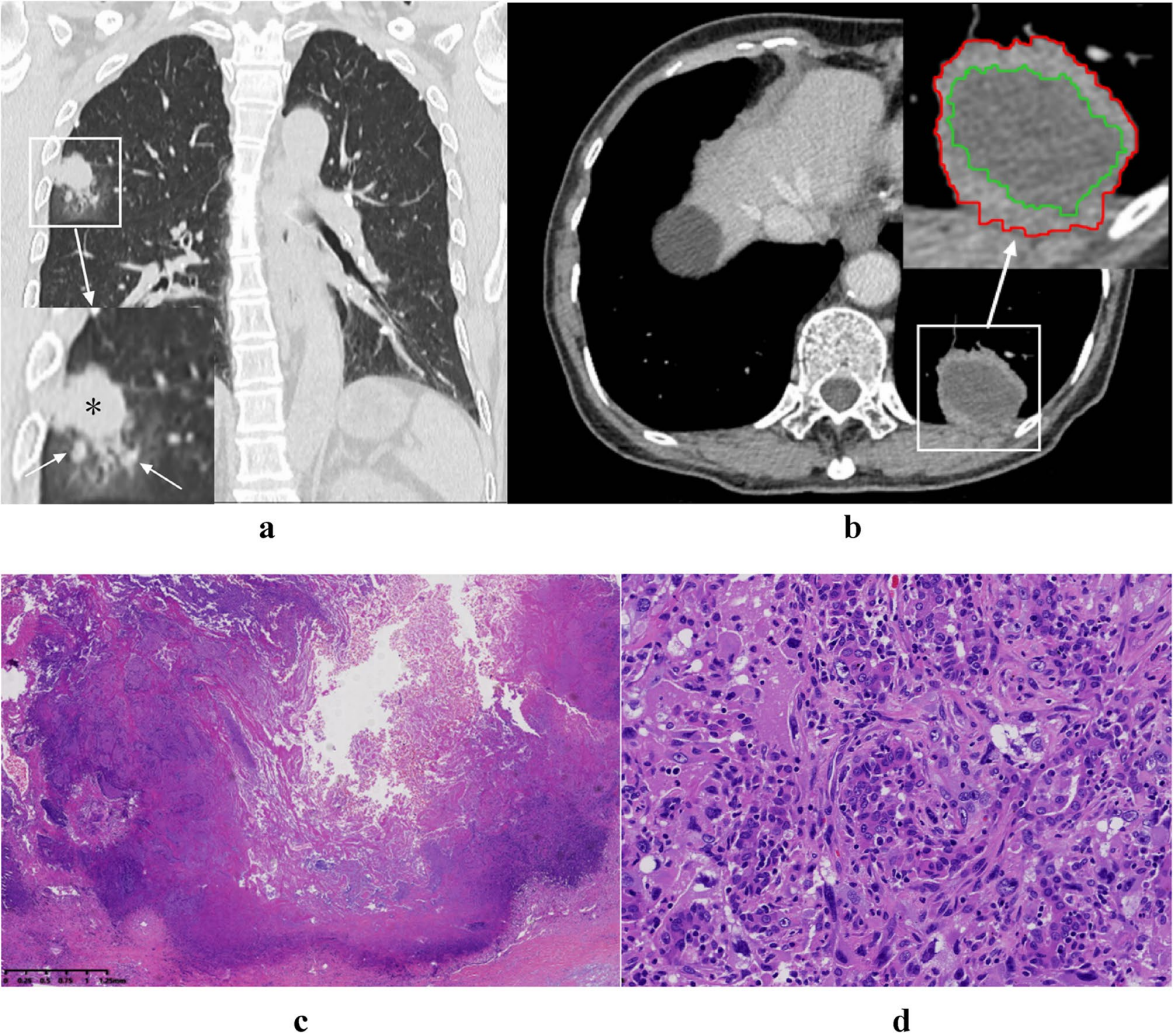
CT and pathological findings of s-NSCLC. **a** A 54-year-old male patient presented with GGO and multiple nodules (arrows in the enlarged image) below the s-NSCLC lesion (*) in the upper lobe of the right lung. The patient died at 8 months of follow-up. **b-c** A 68-year-old female patient with stage T2aN0M0 disease who died after 14 months of follow-up. **b** The LAA ratio was defined as the percentage of the largest low-attenuation area (green ring) to the largest tumor area (red ring). In this case, the LAA ratio was 61%. **c** Low-magnification histopathological examination revealed extensive coagulative necrosis in the tumor center. (HE × 20). **d** High-magnification histopathological examination of the tumor lesion revealed adenocarcinoma cells with mesenchymal transformation (HE × 200)

In this study, three radiologists (WT, YY and SL) with more than 8 years of experience in chest CT diagnosis retrospectively reviewed the CT images and evaluated the tumor size, tumor location, calcification, vacuole/cavity, hydrothorax, LAA ratio and peritumoral GGO or nodules. GGO is defined as a hazy area of increased lung attenuation that does not obscure the underlying bronchial structures or pulmonary vessels, with preserved visibility of parenchymal architecture; a nodule is defined as a well- or poorly marginated rounded or irregular opacity, solid or subsolid, measuring ≤ 3 cm in diameter, surrounded by pulmonary parenchyma (Fig. 1a). Lung window (WW 1500 Hu and WL −500 Hu) and mediastinal window (WW 350 Hu and WL 40 Hu) images were used for analysis.

In the largest tumor layer, the largest tumor size was measured. The parameters measured by the three radiologists were averaged. According to previous studies [8, 11, 12], LAA can be characterized by liquid density (from 0 to 20 Hu) or equal density on plain scans with no enhancement, whereas the peripheral solid component is significantly enhanced. Regions of interest (ROIs) of the largest tumor area and largest LAA were outlined in the VP sequence. The LAA ratio was defined as the percentage of the LAA area to the tumor area (Fig. 1b-d).

The final result was the mean value measured by three radiologists. An interobserver agreement analysis was performed on the CT interpretations by three radiologists, all of whom were blinded to the pathological results. In cases of disagreement, three radiologists reached a consensus through discussion.

### Statistical analysis

The statistical analysis was performed using SPSS version 23.0 (IBM Corp, Armonk, NY, USA) and R software (version 4.2.1, https://www.r-project.org/) for Windows. Normally distributed data are expressed as the mean ± standard deviation; otherwise, they are expressed as the median (interquartile range, IQR: M_p25_, M_p75_). The intraclass correlation coefficient (ICC) and Kendall’s W test were used to evaluate the interobserver agreement of the CT interpretations. The Kaplan‒Meier method was used to calculate the median survival time. The OS of s-NSCLC patients was analyzed via log-rank tests. Prognostic risk factors associated with OS were evaluated by a multivariate Cox regression model. *P* < 0.05 was considered to indicate statistical significance. Nomogram construction was employed to evaluate the predictive factors associated with OS.

## Results

### Clinical and CT findings of s-NSCLC

A total of 135 patients with s-NSCLC confirmed by postoperative pathology (55 patients) and needle biopsy (80 patients) were included. There were 108 males and 27 females among the s-NSCLC patients. The age of the patients in the s-NSCLC group was 62 ± 10 years (range 29–88 years). A total of 108 patients with s-NSCLC underwent enhanced CT scans.

The mean tumor size was 5.8 ± 2.6 cm (range 2.0–14.5 cm). Smoking status accounted for 68.1% (92/135) of the s-NSCLC patients. Ninety-eight cases were located in the periphery, and 37 cases were located in the central region. Calcification (19/135, 14.1%) and vacuole/cavity (22/135, 16.2%) were rare in s-NSCLC lesions. Among the 135 s-NSCLC patients, 36/135 (26.7%) had hydrothorax. Peritumoral GGO or nodules were present in 31/135 (23.0%) patients.

Among the 108 patients who underwent enhanced CT scans, 84 (77.8%) presented with LAA. The LAA ratio of s-NSCLC patients was 30.8% (IQR: 10.6%, 50.7%). Three radiologists conducted interobserver agreement tests on the CT findings, the ICCs were 0.810–0.956, and Kendall’s W coefficients were 0.877–0.981 (*P* < 0.001).

### TNM classification

Among the s-NSCLC patients, 8 had stage I disease according to the TNM classification, 20 had stage II disease, 59 had stage III disease and 48 had stage IV disease. The tumor status was T1 in 4 patients (T1c), T2 in 38 patients, T3 in 38 patients and T4 in 55 patients. The nodal status was N0 in 40 patients, N1 in 7 patients, N2 in 58 patients, and N3 in 30 patients. M1 status was detected in 48 patients, whereas M0 status was detected in 87 patients.

### Long-term survival and Cox regression analysis

**Fig. 2:**
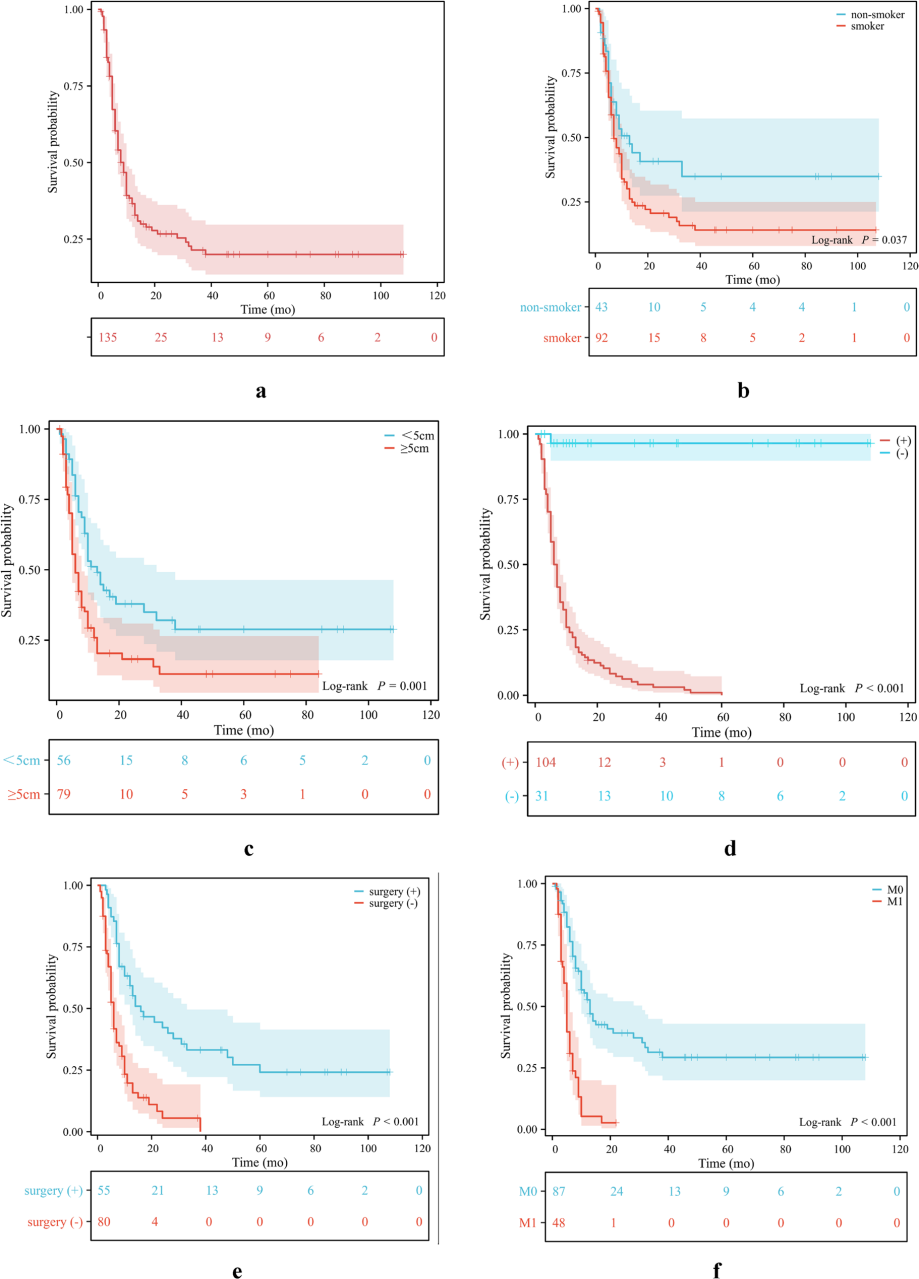
Kaplan‒Meier overall survival curves for s-NSCLC patients (**a**), stratified by smoking status (**b**), tumor size **(c**) peritumoral GGO or nodules (**d**), surgery (**e**) and M stage (**f**)

The median survival time of patients with s-NSCLC was 9 months (95% CI: 7, 11). The 1-year, 2-year and 3-year OS rates for s-NSCLC patients were 36.6%, 26.7% and 21.4%, respectively. (Fig. 2a)

**Fig. 3:**
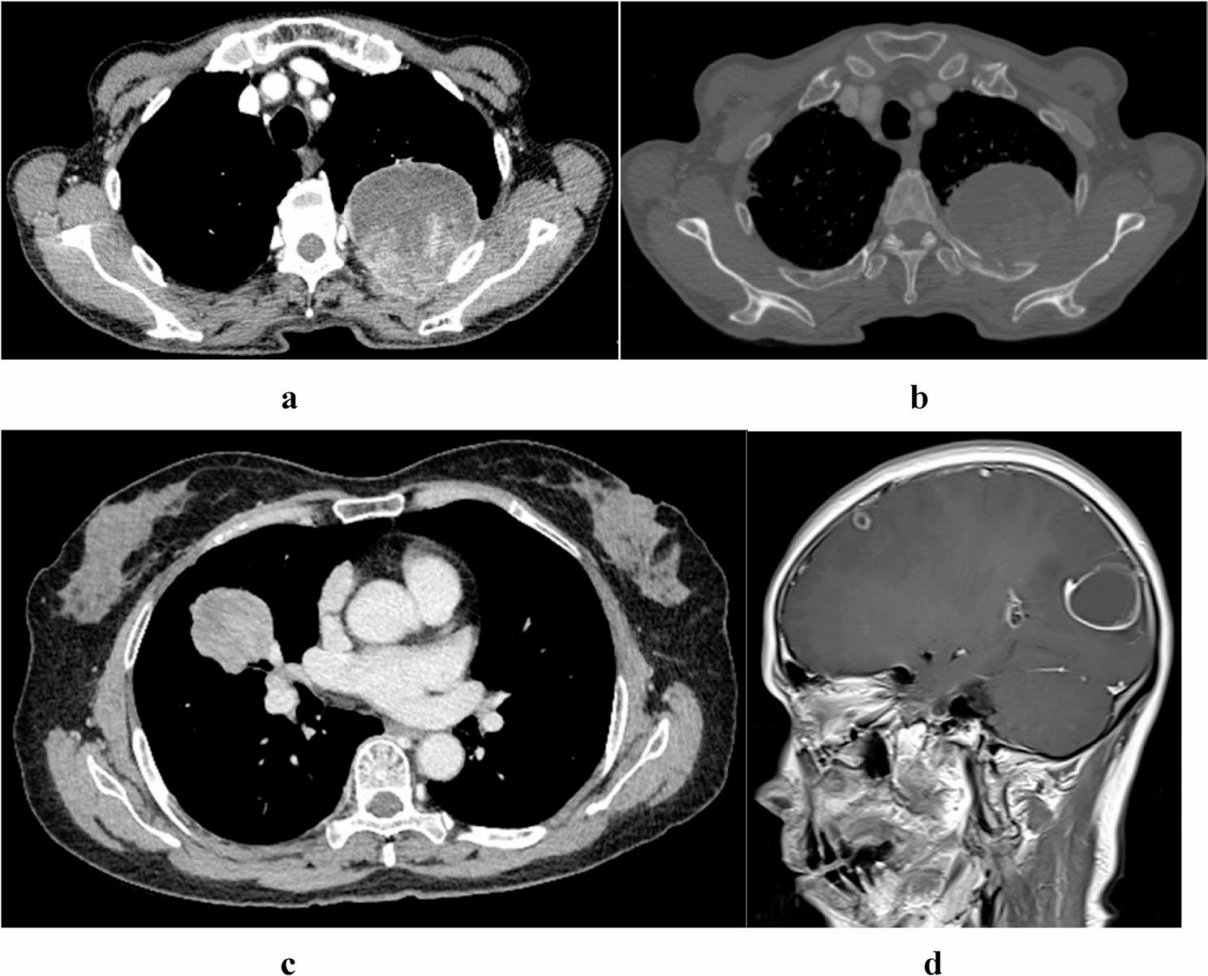
Case reports showing that the LAA ratio is not associated with OS in s-NSCLC patients. **a**-**b** A 79-year-old man with a lesion in the left upper lobe of the lung invading the soft tissue of the chest wall and adjacent ribs. The lesion had a large LAA ratio (51.6%). Complete resection of the lesion and extended resection of the adjacent ribs were performed. The postoperative stage was T4N0M0. The patient was still alive at the 75-month follow-up. **c**-**d** A 53-year-old woman with s-NSCLC located peripherally in the upper lobe of the right lung. The tumor lesion in the venous phase showed heterogeneous and delayed enhancement without the LAA. However, this patient presented with multiple craniocerebral metastases, stage T2bN0M1c. The patient died at 17 months of follow-up

In the univariate analysis, smoking status (*P* = 0.037), tumor size (*P* = 0.001), calcification (*P* = 0.021), peritumoral GGO or nodules (*P* < 0.001), hydrothorax (*P* < 0.001), surgery (*P* < 0.001), overall stage (*P* < 0.001), T factor (*P* < 0.001), N factor (*P* = 0.006) and M factor (*P* < 0.001) were significantly associated with OS in s-NSCLC patients, whereas sex (*P* = 0.192), age (*P* = 0.090), location (*P* = 0.063), cavity or vacuole (*P* = 0.088), and PET/CT SUV_max_ (*P* = 0.711) were not associated with OS. The LAA ratio was not associated with the prognosis of s-NSCLC patients (*P* = 0.211) (Fig. 3a-d).

The twelve characteristics (*P* < 0.10) were included in the multivariate Cox model. Smoking status (*P* = 0.034, HR = 1.668 [95% CI: 1.040, 2.678]), tumor size (*P* = 0.008, HR = 1.818 [95% CI: 1.167, 2.832]), peritumoral GOO or nodules (*P* = 0.026, HR = 2.064 [95% CI: 1.090, 3.909]), surgery (*P* = 0.005, HR = 0.467 [95% CI: 0.274, 0.797]), and M stage (*P* < 0.001, HR = 2.479 [95% CI: 1.476, 4.164]) were found to be independently associated with prognosis (Fig. 2b-f). Age (*P* = 0.789), location (*P* = 0.352), calcification (*P* = 0.568), cavity or vacuole (*P* = 0.992), hydrothorax (*P* = 0.986), T factor (*P* = 0.605), and N factor (*P* = 0.347) were not independent risk factors for s-NSCLC (Table 1).

**Table 1:** Univariate and multivariate analyses of overall survival in s-NCLCS patients

Characteristic	Univariate		Multivariate			
	*n*	*P *value	*P *value	*β*	*HR*	*95% CI*
Clinical characteristics						
Sex (male vs. female)	108 vs. 27	0.192				
Age (< 60y vs. ≥ 60y)	50 vs. 85	0.090	0.789			
Smoking status (- vs. +)	43 vs. 92	0.037	0.034^*^	0.512	1.668	1.040-2.678
Radiological findings						
Size (< 5 cm vs. ≥5 cm)	56 vs. 79	0.001	0.008^**^	0.598	1.818	1.167-2.832
Location (peripheral vs. central)	98 vs. 37	0.063	0.352			
Calcification (- vs. +)	116 vs. 19	0.021	0.568			
Cavity or vacuole (- vs. +)	113 vs. 22	0.088	0.992			
LAA (- vs. +)	24 vs. 84	0.211				
LAA ratio (＜10% vs. 10-50% vs. >50%)	26 vs. 54 vs. 28	0.360				
Peritumoral GGO or nodules (- vs. +)	104 vs. 31	< 0.001	0.026^*^	0.725	2.064	1.090-3.909
Hydrothorax (- vs. +)	99 vs. 36	< 0.001	0.986			
Treatment						
Surgery (- vs. +)	80 vs. 55	< 0.001	0.005^**^	-0.761	0.467	0.274-0.797
TNM classification						
T stage (T1-2 vs. T3 vs. T4)	42 vs. 38 vs. 55	< 0.001	0.605			
N stage (- vs. +)	40 vs. 95	0.006	0.347			
M stage (- vs. +)	87 vs. 48	< 0.001	< 0.001^***^	0.908	2.479	1.476-4.164

*n *number of patients, *NSCLC *Sarcomatoid non-small cell lung cancer, *HR *Hazard ratio, *CI *Confidence interval, *LAA *Low-attenuation area, *GGO *Ground-glass opacity, *TNM *Tumor-node-metastasis

^***^P < 0.001; ^**^P < 0.01;^*^P < 0.05

**Fig. 4:**
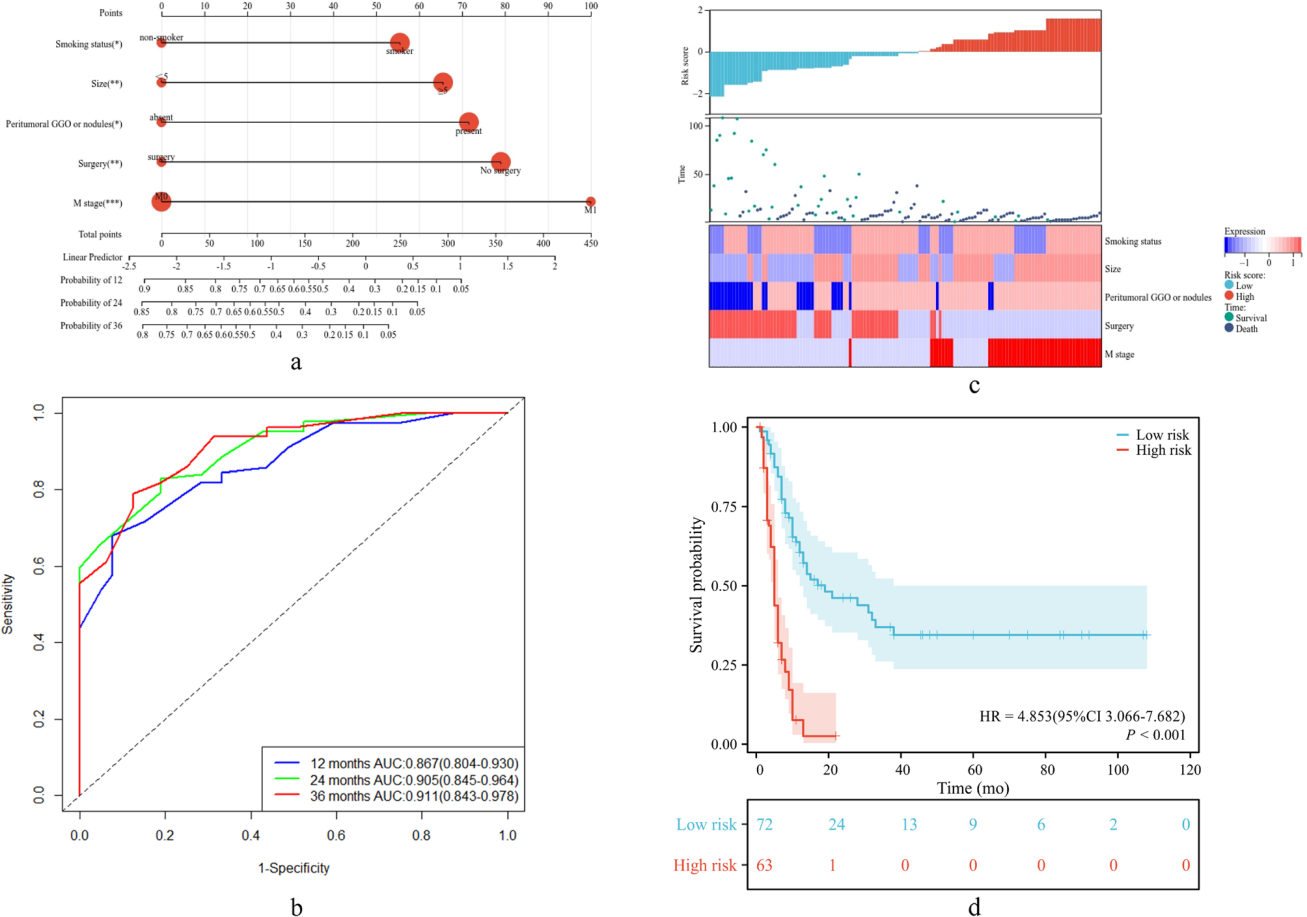
Multivariate Cox regression model for predicting the 12-, 24-, and 36-month OS rates and risk stratification of s-NSCLC patients. **a** Nomogram, **b** ROC curve, and **c** relationship between the prognostic model and clinicoradiological factors in patients with s-NSCLC. **d** Overall survival curves stratified by high- and low- risk groups

The predictors incorporated into the nomogram included smoking status, tumor size, peritumoral GGO or nodules, surgery and M stage, which were statistically significant in multivariate Cox regression analysis. Risk-score = 0.512×smoking status + 0.598×tumor size + 0.725×peritumoral GGO or nodules + (−0.761)×surgery + 0.908×M stage. The optimal cutoff value for risk stratification was determined to be 1.237. We constructed a nomogram for predicting the 12-, 24-, and 36-month OS rates of s-NSCLC patients, and the AUCs were 0.867 (95% CI 0.804–0.930), 0.905 (95% CI 0.845–0.964) and 0.911 (95% CI 0.843–0.978), respectively (Fig. 4a-b). The overall survival curves based on the risk-score were stratified into high- and low-risk groups. In the high-risk group, the median survival time was 5 months, whereas in the low risk group, the median survival time was 19 months (*P* < 0.001, HR = 4.853 [95% CI: 3.066, 7.682]) (Fig. 4c-d).

## Discussion

The name and origin of s-NSCLC have been debated for decades. According to the latest WHO classification of lung neoplasms in 2021, s-NSCLC can be categorized into three pathological subtypes: pleomorphic carcinoma (formed by spindle or/and giant cells), carcinosarcoma, and pulmonary blastoma [13]. The pleomorphic carcinoma subtypes have striking immunohistochemistry and molecular pathology similarities with conventional NSCLC (e.g., adenocarcinoma and squamous cell carcinoma), and their mesenchymal components express epithelial IHC markers, supporting the notion that s-NSCLC originates from epithelial and dedifferentiated variants of conventional NSCLC [14–16]. The sarcomatous components of carcinosarcoma are real sarcomas, and IHC analysis of the sarcomatous components does not reveal the expression of epithelial markers [14–16]. Dual-stage pulmonary blastomas are neoplasms composed of embryonic epithelial elements and primitive mesenchymal stroma. Although carcinosarcoma and pulmonary blastoma have biphasic histological components with separate IHC markers, recent studies have shown that both components have the same genetic alterations, strongly suggesting that the tumors are of monoclonal origin and are differentiated from totipotent tumor stem cells that undergo sarcomatous transformation [14, 17, 18].

Our study revealed that s-NSCLC patients were more likely to be male smokers, with a male-to-female ratio of 4:1. Among the s-NSCLC patients, the mean age at diagnosis was 62 years, and the mean tumor size was 5.8 cm. The median survival time of s-NSCLC patients was only 9 months (95% CI: 7, 11), and the 1-, 2- and 3-year OS rates for s-NSCLC patients were 36.6%, 26.7% and 21.4%, respectively. Our clinical findings are similar to those of previous studies [3, 4, 11, 18, 19], which revealed that the majority of s-NSCLC patients were diagnosed at an advanced stage, leading to missed opportunities for surgical treatment. Furthermore, most s-NSCLC patients (81/135, 60%) in this study died within one year, indicating that this highly malignant tumor progresses rapidly, recurs postoperatively and metastasizes early, leading to short-term mortality.

Our study revealed that smoking is an independent risk factor for the overall survival of s-NSCLC patients. Nonsmokers are characterized by a lower burden of tumor mutations and no mutational signatures associated with tobacco exposure [20]. Smoking may induce genomic instability in tumor cells, leading to sarcomatoid metaplasia, which is associated with poor prognosis. Our study revealed that surgical treatment was independently associated with better survival, which is consistent with the findings of previous studies [4, 21]. These findings suggest that complete surgical resection is essential for the long-term survival of patients with s-NSCLC. Our study revealed that 48/135 (35.6%) of the s-NSCLC patients had distant metastasis at diagnosis. Furthermore, M stage and tumor size were found to be independent risk factors for prognosis. This highlights the importance of early diagnosis and timely intervention in s-NSCLC patients.

Our study revealed that the majority of s-NSCLC lesions presented with LAA (87 of 108, 80.6%). Kim et al. [8] reported that 8/10 (80%) s-NSCLC lesions had LAA lesions. Fujisaki et al. [11] reported the presence of LAA in 40/44 (91%) s-NSCLC patients, which was similar to the findings of our study. Furthermore, our study revealed that the median LAA ratio was 30.8% in s-NSCLC patients. The pathological manifestations of LAA are mucinous degeneration, necrosis, and hemorrhage, suggesting that rapid proliferation exceeds the blood supply [8, 10]. In our study, CT findings revealed that calcification (19 of 135, 14.1%) and vacuole/cavity (22 of 135, 16.3%) were rare in s-NSCLC patients, which was consistent with the findings of previous studies [10, 19].

In a previous study [11], the LAA ratio was found to be an independent risk factor for s-NSCLC: a LAA ratio > 25% was associated with shorter OS and disease-free survival than a LAA ratio < 25%. A study by Nishida et al. [10] reached a similar conclusion. However, the LAA ratio was not associated with OS in our study. While previous studies evaluating the LAA ratio focused exclusively on surgically resected s-NSCLC patients, our study included predominantly advanced-stage patients. This difference in patient population likely accounts for the lack of association between the LAA ratio and OS in our cohort, as evidenced by the significant heterogeneity in tumor characteristics (e.g., tumor size, extent of necrosis, and overall stage). We further performed separate analyses of surgical cases but also found no significant association between the LAA ratio and prognosis in s-NSCLC patients. This may be because s-NSCLC is prone to metastasis, and these lesions have not yet grown large enough to form LAA when distant metastasis occurs, leading to death in the short term.

In our study, peritumoral GGO or nodules was found to be an independent risk factor associated with poor prognosis in s-NSCLC patients. These distinctive CT findings indicate tumor invasion into surrounding tissues, bronchial involvement, and peritumoral metastasis, all of which are indicative of a high degree of tumor aggressiveness and metastatic potential. Nishida et al. [10] reported that GGO can be observed across all subtypes of s-NSCLC, and the pathological features associated with GGO are hemorrhage, vascular invasion, and aerogenic metastasis. However, the study by Nishida and colleagues did not find a relationship between GGO and prognosis. This could be attributed to the fact that the study focused solely on surgically treated patients, excluding advanced-stage patients with more severe peritumoral invasion. Moreover, the presence of small peritumoral nodules may represent either lymphatic dissemination of s-NSCLC or the development of small metastatic tumor foci, both of which are associated with poorer clinical outcomes.

In the present study, we constructed a nomogram for predicting the 1-, 2-, and 3-year OS rates of s-NSCLC patients. The predictors incorporated into the nomogram included smoking status, tumor size, peritumoral GGO or nodules, surgery and M stage. The nomogram and risk stratification offer two clinical implications. First, it provides a quantitative tool for clinicians to predict the OS of s-NSCLC patients. Moreover, early identification of high-risk individuals can lead to timely interventions. Our research results are consistent with those of Kim et al. [22], who reported that CT and positron emission tomography (PET) features including location, cavity, and maximum standardized uptake value (SUV_max_) combined with radiomics enables effective risk stratification of s-NSCLC patients. Further research is needed to evaluate the prognostic role of CT radiomics and PET in s-NSCLC patients.

This study has several limitations. First, this was a retrospective study and selection bias is inevitable. Second, the histological confirmation of pulmonary sarcomatoid carcinoma mandates that sarcomatoid elements constitute more than 10% of the whole tumor in the surgical specimen. However, owing to the highly aggressive nature of this malignancy, most cases are already at advanced stages upon detection, missing the opportunity for complete surgical resection. Therefore, our study included advanced-stage cases confirmed by needle biopsy. Moreover, we designated our research group as “s-NSCLC”. Third, there is heterogeneity in imaging protocols across different centers. However, this does not significantly affect radiologists’ interpretation of CT imaging features. Future prospective studies with standardized imaging parameters are warranted. Finally, other treatment strategies, such as radiochemotherapy, were not included in our study because of controversy regarding their efficacy in s-NSCLC. Furthermore, this study dates back more than 10 years, and the treatment guidelines have changed greatly over this period. Prospective studies with uniform treatment standards are needed in the future.

In conclusion, the large intratumoral LAA ratio on CT was a characteristic imaging finding of s-NSCLC. However, it is not a prognostic factor for s-NSCLC. Smoking status, tumor size, peritumoral GGO or nodules, surgery and M stage were found to be independent prognostic factors associated with OS in s-NSCLC patients. The nomogram and risk-score are capable of predicting the 1-, 2-, and 3-year OS rates and risk stratification for s-NSCLC patients. These findings are helpful for the CT diagnosis of s-NSCLC and provide a reference for prognostic evaluation in clinical management.

## Data Availability

The datasets used and/or analyzed during the current study are available from the corresponding author on reasonable request.

## Abbreviations

s-NSCLC: Sarcomatoid non-small cell lung cancer
TNM: Tumor-node-metastasis
LAA: Low attenuation area
GGO: Ground-glass opacity
IQR: Interquartile range
CI: Confidence interval
ROI: Region of interest
AUC: Area under the curve
OS: Overall survival
HR: Hazard ratio
